# Evaluation of the Quadri-Planes Method in Computer-Aided Diagnosis of Breast Lesions by Ultrasonography: Prospective Single-Center Study

**DOI:** 10.2196/18251

**Published:** 2020-05-05

**Authors:** Liang Yongping, Zhang Juan, Ping Zhou, Zhao Yongfeng, Wengang Liu, Yifan Shi

**Affiliations:** 1 The Xiangya Medical School Central South University Changsha, Hunan China

**Keywords:** ultrasonography, breast neoplasm, breast imaging reporting and data system (bi-rads), breast neoplasm diagnosis, cancer screening, computer-aided diagnosis, breast cancer

## Abstract

**Background:**

Computer-aided diagnosis (CAD) is a tool that can help radiologists diagnose breast lesions by ultrasonography. Previous studies have demonstrated that CAD can help reduce the incidence of missed diagnoses by radiologists. However, the optimal method to apply CAD to breast lesions using diagnostic planes has not been assessed.

**Objective:**

The aim of this study was to compare the performance of radiologists with different levels of experience when using CAD with the quadri-planes method to detect breast tumors.

**Methods:**

From November 2018 to October 2019, we enrolled patients in the study who had a breast mass as their most prominent symptom. We assigned 2 ultrasound radiologists (with 1 and 5 years of experience, respectively) to read breast ultrasonography images without CAD and then to perform a second reading while applying CAD with the quadri-planes method. We then compared the diagnostic performance of the readers for the 2 readings (without and with CAD). The McNemar test for paired data was used for statistical analysis.

**Results:**

A total of 331 patients were included in this study (mean age 43.88 years, range 17-70, SD 12.10), including 512 lesions (mean diameter 1.85 centimeters, SD 1.19; range 0.26-9.5); 200/512 (39.1%) were malignant, and 312/512 (60.9%) were benign. For CAD, the area under the receiver operating characteristic curve (AUC) improved significantly from 0.76 (95% CI 0.71-0.79) with the cross-planes method to 0.84 (95% CI 0.80-0.88; *P*<.001) with the quadri-planes method. For the novice reader, the AUC significantly improved from 0.73 (95% CI 0.69-0.78) for the without-CAD mode to 0.83 (95% CI 0.80-0.87; *P*<.001) for the combined-CAD mode with the quadri-planes method. For the experienced reader, the AUC improved from 0.85 (95% CI 0.81-0.88) to 0.87 (95% CI 0.84-0.91; *P*=.15). The kappa indicating consistency between the experienced reader and the novice reader for the combined-CAD mode was 0.63. For the novice reader, the sensitivity significantly improved from 60.0% for the without-CAD mode to 79.0% for the combined-CAD mode (*P*=.004). The specificity, negative predictive value, positive predictive value, and accuracy improved from 84.9% to 87.8% (*P*=.53), 76.8% to 86.7% (*P*=.07), 71.9% to 80.6% (*P*=.13), and 75.2% to 84.4% (*P*=.12), respectively. For the experienced reader, the sensitivity improved significantly from 76.0% for the without-CAD mode to 87.0% for the combined-CAD mode (*P*=.045). The NPV and accuracy moderately improved from 85.8% and 86.3% to 91.0% (*P*=.27) and 87.0% (*P*=.84), respectively. The specificity and positive predictive value decreased from 87.4% to 81.3% (*P*=.25) and from 87.2% to 93.0% (*P*=.16), respectively.

**Conclusions:**

S-Detect is a feasible diagnostic tool that can improve the sensitivity, accuracy, and AUC of the quadri-planes method for both novice and experienced readers while also improving the specificity for the novice reader. It demonstrates important application value in the clinical diagnosis of breast cancer.

**Trial Registration:**

ChiCTR.org.cn 1800019649; http://www.chictr.org.cn/showproj.aspx?proj=33094

## Introduction

Breast cancer is one of the most common cancers in women and the second leading cause of cancer-related mortality worldwide [1,2]. Early diagnosis of breast cancer can increase the treatment options and survival rate of patients [3]; however, early diagnosis depends on accurate and reliable diagnosis using medical imageology. As a convenient modality, breast ultrasonography plays an important role in breast cancer screening. Despite the improvements in ultrasound diagnosis with the application of new technology, dependence on operator experience remains the main limitation of ultrasound-based diagnosis [4,5]. S-Detect is a recently developed computer-aided diagnosis (CAD) system for breast cancer that provides assistance in morphological analysis based on the Breast Imaging Reporting and Data System (BI-RADS) lexicon and classification [6]. Many studies have reported that the S-Detect system has potential to become a novel diagnostic tool for radiologists [7-10].

In our previous study, the sensitivity was too high in the cross-planes method because it considered the lesion to be malignant if any image of 2 planes indicated malignancy, leading to a decrease in specificity. No study has evaluated the diagnostic performance of CAD in breast lesions with respect to diagnostic planes (cross-plane and quadri-plane methods). Therefore, the purpose of this study was to compare the performance of radiologists with different levels of experience in detecting breast cancer using CAD with the quadri-planes method.

## Methods

### Patient Selection

We prospectively enrolled patients in our study from November 2018 to October 2019. All patients underwent grayscale breast ultrasound examination before surgery. All lesions were examined after surgery to confirm their pathological type. This prospective single center study was approved by the Institutional Review Board of Third Xiangya Hospital. Informed consent was obtained from all patients.

The inclusion criteria were as follows: patients aged 17-70 years with breast tumors requiring surgery. The exclusion criteria were a history of neoadjuvant chemotherapy or endocrine therapy before surgery, lesions punctured by core-needle biopsy or a Mammotome system, equipment of the breast with a prosthesis, unclear lesions as displayed by ultrasound images, and unwillingness to take part in the study.

### Ultrasound Image Acquisition

All images were obtained with an RS80A ultrasound system (Samsung Medison Co Ltd) with a 5-13 megahertz bandwidth (8.4 MHz center frequency) linear transducer. All ultrasound examinations were performed by an independent radiologist with 5 years of experience. In the cross-planes method, 2 typical images of the tumor in the longitudinal and transverse planes were stored in the ultrasound system; in the quadri-planes method, 2 additional cross-plane images were acquired by rotating the probe 45 degrees around the center of the mass.

### Computer-Aided Diagnostic System

Our CAD system, S-Detect, extracts features using an integration of artificial neural network classifiers internally installed in the RS80A ultrasound equipment. The sensitivity of the instrument was set to the default. To test the reproducibility of the CAD marks with the same image, we randomly selected 20/512 (3.9%) examinations and passed them through the CAD system 3 times; the results showed that the markings were consistent in all images.

In S-Detect, the cursor was placed on the identified center of the lesion, and a region of interest was automatically drawn along the border of the mass by the ultrasound system. If the borderline was considered inaccurate in any area of the tumor, it was manually edited to achieve the optimum fitness. The ultrasound image features of the lesion were analyzed according to the BI-RADS lexicon, and the final classifications were automatically performed by the ultrasound system. In the S-Detect system, the final assessment classification was divided into dichotomous results of “possibly benign” or “possibly malignant.”

### Diagnostic Criteria

According to the fifth version of BI-RADS, the radiologists classified the lesions from category 3 to category 5. BI-RADS category 4 was further subdivided into categories 4A, 4B, and 4C. Category 3 is considered probably benign (<2% likelihood of malignancy), and categories 4A, 4B, and 4C range from low to high suspicion (2%-10%, 10%-50%, and 50%-95% likelihood of malignancy, respectively). Category 5 indicates a high malignancy rate (>95% likelihood of malignancy). Malignant signs in breast ultrasound imaging include irregular shape, antiparallel orientation, noncircumscribed margin, microcalcification, acoustic halo, posterior shadowing, and abnormalities of the surrounding tissue. Lesions with no definitive malignant sign were assigned to category 3; lesions with 1, 2, and 3 malignant signs were assigned to categories 4A, 4B, and 4C, respectively; and lesions with more than 4 malignant signs were assigned to category 5. Accordingly, category 3 and 4A lesions were regarded as benign, and category 4B, 4C, and 5 lesions were regarded as malignant [11,12].

To assess the combination of ultrasound and the CAD system, we acquired images of the longitudinal and transverse planes of the tumor for CAD with the cross-planes method. If 1 plane indicated “possibly malignant,” the outcome was considered positive, and the BI-RADS category diagnosis was increased by 1 level (ie, 3 to 4A, 4A to 4B, 4B to 4C, 4C to 5). If both planes indicated “possibly benign,” the outcome was considered negative, and the BI-RADS category diagnosis was decreased by 1 level (ie, 5 to 4C, 4C to 4B, 4B to 4A, 4A to 3) [13]. For the quadri-planes method, if any 2 planes indicated “possibly malignant,” the outcome was considered positive, and the BI-RADS category diagnosis was increased by 1 level. If all 4 planes indicated “possibly benign,” the outcome was considered negative, and the BI-RADS category diagnosis was decreased by 1 level.

### Readers, Reading Modes, and Training

The study included 2 readers: a novice reader with 1 year of ultrasound experience and an experienced reader with 5 years of ultrasound experience. Both readers were trained in the reading procedures with 20 ultrasound images (from the 512 examinations) that were not part of the study set, 10 of which were read without using CAD (without-CAD mode). The readers assessed the other 10 images in combined-CAD mode with the cross-planes method and the quadri-planes method; the readers first read the ultrasound images without using CAD and then mechanically combined the indications of the CAD marks to make the final decision.

**Figure 1:**
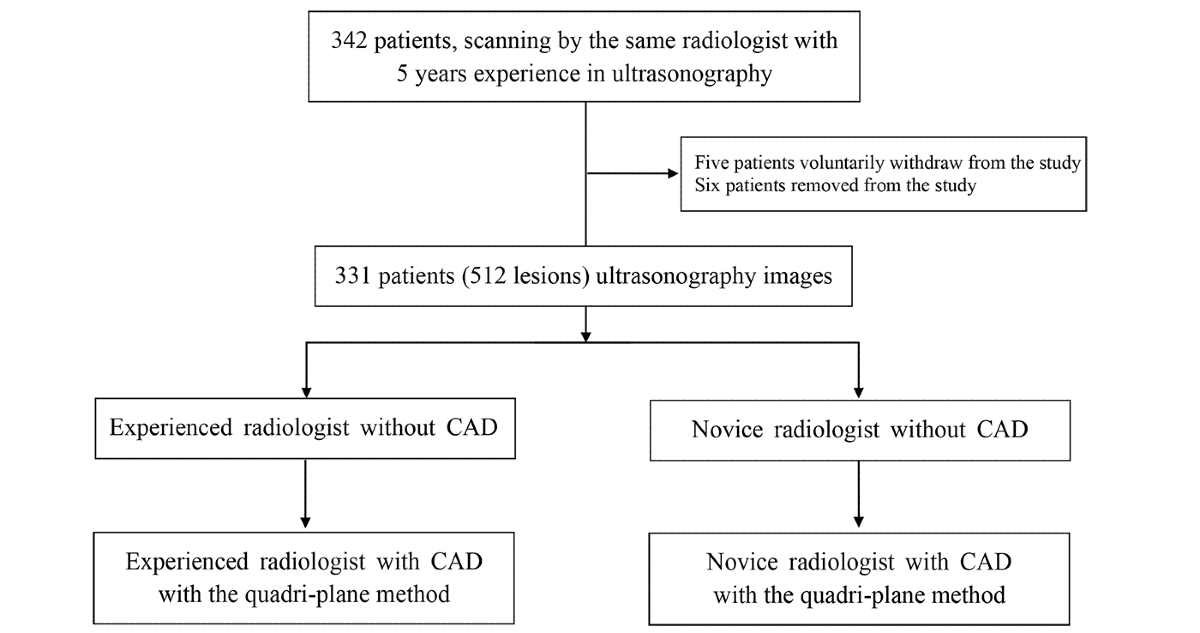
The study design and workflow. CAD: computer-aided diagnosis.

Both readers performed every examination in each reading mode independently and were blinded to any information about the patients, including age, manifestation of symptoms, and previous radiology reports. The readers were asked to read for at least 2 hours per day to simulate the typical process of batch reading in such examinations (Figure 1).

### Statistical Analysis

Statistical evaluation was performed using SPSS software version 19.0 (IBM Corporation). Taking the pathology results as the gold standard, we analyzed the diagnostic sensitivity, specificity, and area under the receiver operating characteristic (ROC) curve (AUC) in without-CAD mode and combined-CAD mode (with the quadri-planes method). The confirmatory diagnosis was defined as the diagnosis made on the basis of pathology. The diagnostic parameters of the combined-CAD mode and without-CAD mode were compared using the McNemar test for sensitivity, specificity, positive predictive value (PPV), negative predictive value (NPV), and accuracy for match-paired data. We used the Hanley and McNeil method to analyze the differences between pairs of AUCs. The number of malignant planes of each tumor was recorded with the quadri-planes method, and the ROC curves were drawn based on the pathological results to determine the cutoff value based on the maximum Youden index. The degree of agreement between the experienced reader in without-CAD mode and the novice reader in combined-CAD mode was analyzed using kappa statistics. The criteria for the kappa values were poor ≤0.2, fair 0.21-0.4, moderate 0.41-0.6, good 0.61-0.8, and perfect 0.81-1 [14]. For all tests mentioned, *P*<.05 was considered to indicate statistical significance.

## Results

### Characteristics of the Patients and Lesions

The patient demographics and lesion characteristics are summarized in Table 1. A total of 331 patients who presented with 512 lesions were included in this study. The mean age of the examined patients was 43.88 years, range 17-70 (SD 12.10). The diameters of the lesions ranged from 0.26-9.50 centimeters, mean 1.85 (SD 1.19). Among the 512 breast lesions, 200/512 (39.1%) were malignant and 312/512 (61.9%) were benign. The mean sizes of all lesions were similar and were close to 2 cm; benign lesions were the smallest (1.82 cm) and malignant lesions were the largest (2.28 cm).

**Table 1:** Patient demographics (N=331) and lesion characteristics (N=512).

Characteristic			Value
**Age (years)**			
	Mean (SD)		43.88 (12.10)
	Median (range)		45 (17-70)
**Age distribution (years), n (%)**			
	<30		39 (11.8)
	30-39		76 (23.0)
	40-49		104 (31.4)
	50-59		80 (24.2)
	60-70		32 (9.7)
**Lesion size (cm)^a^**			
	Mean (SD)		1.85 (1.19)
	Median (range)		1.7 (0.26-9.50)
**Malignant lesion size (cm)**			
	Mean (SD)		2.28 (1.10)
	Median (range)		2.18 (0.26-6.20)
**Benign lesion size (cm)**			
	Mean (SD)		1.82 (1.39)
	Median (range)		1.41 (0.37-9.50)
**Pathological finding, n (%)**			
	Malignant		200 (39.1)
	Benign		312 (60.9)
**Histological type, n (%)**			
	**Malignant (n=200)**		
		Intraductal carcinoma in situ	9 (4.5)
		Invasive lobular carcinoma	17 (8.5)
		Mucinous adenocarcinoma	4 (2.0)
		Medullary carcinoma	3 (1.5)
		Invasive ductal carcinoma	167 (83.5)
	**Benign (n=312)**		
		Intraductal papilloma	37 (11.9)
		Granulomatous mastitis	8 (2.6)
		Fibroma	211 (67.6)
		Hyperplasia-induced lesions	5 (17.31)
		Scar tissue	2 (0.6)

^a^cm: centimeters.

### Reader Performance

The diagnostic performance of CAD and of the novice and experienced readers in comparison with the pathological diagnoses is depicted in Table 2.

The statistical evaluation of the performance of the CAD system and of the readers is shown in Table 2. For CAD, the AUC improved significantly between the cross-planes method and the quadri-planes method (*Z*=4.42, *P*<.001). The cutoff value of the positive planes in the quadri-planes method was 2.5 based on the Youden index of 0.68. Considering that breast cancer often demonstrates invasive characteristics and has relatively poor prognosis [15], we set the threshold to any 2 positive planes of 4 images.

**Figure 2:**
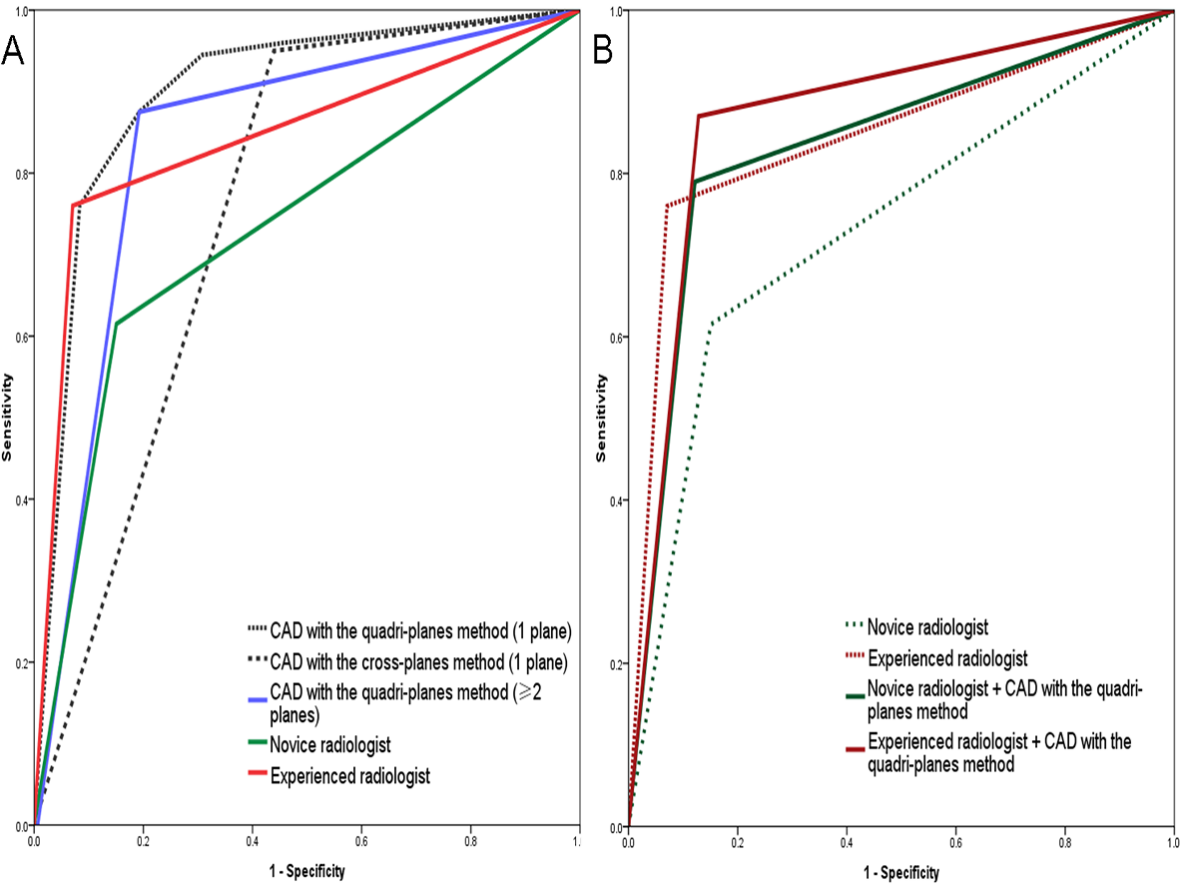
Receiver operating characteristic curves for the CAD method and the readers. (A) CAD with the quadri-plane and cross-plane methods (if either plane was malignant, the result was regarded as positive); CAD with the quadri-planes method with 2 planes as the threshold (if ≥2 planes were malignant, the result was regarded as positive); the novice reader without CAD; the experienced reader without CAD. (B) The novice reader without CAD; the experienced reader without CAD; the novice reader combined with CAD with the quadri-planes method; the experienced reader combined with CAD with the quadri-planes method. AUC: area under the curve; CAD: computer-aided diagnosis.

For the novice reader, the improvement in the AUCs was significant between the without-CAD mode and combined-CAD mode with the quadri-planes method (*Z*=5.55, *P*<.001). However, there was no significant difference in the AUCs for the without-CAD and combined-CAD modes for the experienced reader (*Z*=1.44, *P*=.15; Table 3, Figure 2). The kappa indicating consistency between the experienced reader in without-CAD mode and the novice reader in combined-CAD mode was 0.63.

When a BI-RADS category 4A threshold was used, in contrast to CAD with the cross-planes method, significant improvements in specificity (*P*<.001), PPV (*P*=.01), and accuracy (*P*=.03) were observed for the quadri-planes method; however, there was no significant difference in NPV, and the sensitivity decreased. The sensitivity, NPV, and accuracy improved in the combined-CAD mode compared with the without-CAD mode for both readers (Table 3). Among these, the sensitivity improved significantly between the 2 reading modes for both the novice reader (*P*=.004) and the experienced reader (*P*=.045), whereas the accuracy improved significantly only for the novice reader. Moreover, there were no significant differences between modes for either reader with respect to specificity, PPV, or NPV.

**Table 2:** Diagnostic performance of the computer-aided diagnosis system and the novice and experienced readers in the 2 reading modes with the Breast Imaging Reporting and Data System Category 4A threshold. The pathological diagnosis was considered to be the gold standard.

Pathological diagnosis	CAD^a^				Novice reader				Experienced reader			
	Cross-planes method		Quadri-planes method		Without-CAD mode		Combined-CAD mode with quadri-planes		Without-CAD mode		Combined-CAD mode with quadri-planes	
	+^b^	–^c^	+	–	+	–	+	–	+	–	+	–
+	190	10	175	25	120	80	158	42	152	48	174	26
–	137	175	58	254	47	265	38	274	22	290	40	272

^a^CAD: computer-aided diagnosis.

^b^+: positive diagnosis. Breast Imaging Reporting and Data System assessment categories 4B, 4C, and 5 were considered positive for cancer.

^c^–: negative diagnosis.

**Table 3:** Statistical evaluation of the performance of the computer-aided diagnosis system and the 2 readers with *P* values indicting differences between various groups.

Characteristic	CAD^a^		Novice reader		Experienced reader		Significance				
	CP^b^ method	QP^c^ method	Without-CAD mode	Combined-CAD mode with QP	Without-CAD mode	Combined-CAD mode with QP	*P* value^d^	*P* value^e^	*P* value^f^	*P* value^g^	*P* value^h^
Sensitivity, %	95.0	87.5	60.0	79.0	76.0	87.0	.048	.004	.045	.61	.04
Specificity, %	56.1	81.4	84.9	87.8	93.0	87.2	<.001	.53	.15	.23	.01
PPV^i^, %	58.1	75.1	71.9	80.6	87.4	81.3	.01	.13	.25	.25	.03
NPV^j^, %	94.6	91.0	76.8	86.7	85.8	91.3	.27	.07	.27	.84	.27
Accuracy, %	71.3	83.8	75.2	84.4	86.3	87.1	.03	.03	.32	.69	.69
AUC^k^	0.76	0.84	0.73	0.83	0.85	0.87	<.001	<.001	0.15	.58	.76

^a^CAD: computer-aided diagnosis.

^b^CP: cross-planes.

^c^QP: quarter-planes.

^d^*P* for CAD with the cross-planes method vs CAD with the quadri-planes method.

^e^*P* for the novice reader without CAD vs the novice reader using CAD with the quadri-planes method.

^f^*P* for the experienced reader without CAD vs the experienced reader using CAD with the quadri-planes method.

^g^*P* for the novice reader using CAD with the quadri-planes method vs the experienced reader without CAD.

^h^*P* for CAD with the quadri-planes method vs the experienced reader without CAD.

^i^PPV: positive predictive value.

^i^NPV: negative predictive value.

^k^AUC: area under the receiver operating characteristic curve.

### Management of Diagnostic Decision Changes

In contrast to the mode without CAD, changes in the diagnostic decision with combined-CAD mode were moderately more common for the novice reader than for the experienced reader (115/512, 22.5% vs 70/512, 13.7%; *P*=.09). The proportions of malignancy lesions correctly upgraded from category 4A to 4B by the novice reader and experienced reader were similar (44/115, 38% vs 27/70, 39%; *P*=.88). However, the proportion of benign lesions correctly downgraded from category 4B to 4A by the novice reader was higher than that by experienced reader, with a very close to significant statistical difference (37/115, 32% vs 10/70, 14%; *P*=.05). The proportions of malignancy lesions incorrectly downgraded from category 4B to 4A by both readers were similar (6/115, 5% vs 5/70, 7%). In addition, the proportion of benign lesions incorrectly upgraded by the novice reader was lower than that by the experienced reader (28/115, 24% vs 28/70, 40%). The management decision changes of the 2 readers are provided in Table 4.

The kappa indicating consistency between the experienced reader and the novice reader with combined-CAD mode was slightly higher than that for without-CAD mode (0.63 vs 0.57). These results are provided in Table 5.

**Table 4:** Management decision changes by the 2 readers using CAD with the quadri-planes method.

Decision change with CAD	Novice reader (n=115)		Experienced reader (n=70)		*P* value	
	Correct, n (%)	Incorrect, n (%)	Correct, n (%)	Incorrect, n (%)	Correct	Incorrect
4A to 4B	44 (38)	28 (24)	27 (39)	28 (40)	.88	<.001
4B to 4A	37 (32)	6 (5)	10 (14)	5 (7)	.05	.47

**Table 5:** Comparisons of consistency between the experienced reader in without-CAD mode and the novice reader in without-CAD mode and combined-CAD mode with the quadri-planes method.

Experienced reader in without-CAD mode	Novice reader in without-CAD^a^ mode^b^		Novice reader in combined-CAD mode^c^	
	+^d^	–^e^	+	–
+	121	53	141	33
–	46	292	55	283

^a^CAD: computer-aided diagnosis.

^b^kappa=0.57.

^c^kappa=0.63.

^d^Positive diagnosis. Breast Imaging Reporting and Data System assessment categories 4B, 4C, and 5 were considered positive for cancer.

^e^Negative diagnosis.

## Discussion

### Principal Findings

In our study, the AUCs of CAD with the quadri-planes method were significantly higher than those of CAD with the cross-planes method (*P*<.001); even when we chose any 2 malignant planes as the threshold, the AUC of the quadri-planes method was still higher than that of the cross-planes method (*P*<.001). The sensitivity, accuracy, and AUC improved for both the novice and experienced readers using combined-CAD mode with the quadri-planes method. Additionally, compared to without-CAD mode, the consistency level improved from fair to good between the novice reader in combined-CAD mode and the experienced reader in without-CAD mode.

Choi et al [10] recently reported that the specificity and AUC of both experienced and inexperienced readers improved using a CAD system combined with S-Detect; moreover, the sensitivity of the inexperienced reader improved significantly. Although the diagnosis performance of the readers improved in Choi’s study, the sensitivity of the readers combined with CAD was not satisfactory for detecting breast cancer (66.7% and 75.0%, respectively). These results may have been obtained because the proportion of malignant lesions was too low (6%); moreover, the data in that study were derived from a small number of patients. All these factors can lead to increased false negative results. According to our previous study [16], high sensitivity is a remarkable characteristic of S-Detect in the cross-planes method; this is similar to some previously published studies, where the sensitivity of the ultrasound CAD system was reported to be high (between 88.9% and 100%) [17,18].

It is known that high sensitivity in diagnostic performance can lead to unnecessary breast biopsies and increased medical costs borne by patients; therefore, we developed the quadri-planes method to address this problem. CAD in the quadri-planes method resulted in both improved sensitivity (60.0% to 79.0%) and specificity (84.9% to 87.8%) for the novice reader; in addition, there was no statistically significant change in specificity for the experienced reader (93.0% to 87.2%), while the sensitivity improved significantly (76.0% to 87.0%). This indicates that CAD with the quadri-planes method can improve the sensitivity and specificity of the results reported by readers, especially less experienced readers.

In our investigation, the specificity, accuracy, and AUC of CAD with the quadri-planes method were all higher than those of CAD with the cross-planes method, although the sensitivity of the quadri-plane method was slightly lower. This is likely because the quadri-planes method is based on the cross-planes method; therefore, 2 of the 4 planes in the quadri-planes method were the same as those in the cross-planes method. In addition, the threshold in the quadri-planes method for dichotomization of the final CAD assessment was set at any 2 of 4 positive planes; however, the threshold in the cross-planes method was set as any 1 of 2 positive planes. This may have led to the increase in specificity and the decrease in sensitivity of the quadri-planes method compared to the cross-planes method.

When the assessments differed in category 4A and 4B between the readers and the CAD, the proportion of correct adjustments using CAD for the inexperienced reader was higher than that for the experienced reader (81/115, 70% vs 37/70, 53%). This indicates that the less experienced reader obtained more benefit from the CAD system; this is related to the fact that combining CAD with the quadri-planes method led to improvements in sensitivity, specificity, and accuracy for the inexperienced reader, while the accuracy between the quadri-planes method and the experienced reader was similar (83.8% vs 86.3%). For CAD with the quadri-planes method, the consistency between the experienced reader and novice reader was good. As such, CAD assistance with the quadri-planes method can not only improve diagnostic performance but can also be expected to play a more weighted role in providing a second opinion, especially for less experienced readers. Consequently, this system can reduce misdiagnosis by less experienced readers in addition to reducing variability in readers’ interpretations and overcoming the effects of inexperience. These improvements in diagnostic performance by combining CAD and ultrasound may reduce both the misdiagnosis and missed diagnosis ratios of breast cancer by readers with different experience levels.

Several reports have described applying different types of CAD to breast ultrasound [6,19-21]. These studies all reported that the CAD systems enhanced the diagnostic performance of breast ultrasound, especially specificity and accuracy. Shen et al [20] argued that CAD systems can be helpful in evaluating fuzzy category 4 lesions. Wang et al [21] suggested that combining CAD is more helpful for inexperienced readers than experienced ones, with greater improvement in the diagnostic performance in the inexperienced group. Kim’s study involved 2 staff radiologists with 7 and 19 years of experience, respectively; both these readers can be described as experienced, so their false positive rates were low and their false negative rates were relatively high. In addition, the surgery proportion was only 27.6% and the core needle biopsy proportion was 61.5%, which may also have affected the results. The retrospective analysis was performed by only 1 radiologist with 7 years of ultrasound experience. In Wang’s study, the CAD system was relatively old, and the experience between the 8 readers varied, which may have led to increases in false negative and false positive rates. Therefore, the results of the above studies show that traditional CAD methods are not sufficient to balance the sensitivity and specificity to effectively reduce false negative or false positive results. In our study, the sensitivity, NPV, and accuracy of both readers improved; this supports the idea that S-Detect can reliably provide a second view that can be referred to by readers. Although the CAD methods were not exactly the same as in previous studies [17,18], high sensitivity balanced with specificity is a remarkable superiority of the quadri-planes method. Instead, the proportion of the benign lesions in our study was lower (Table 1), and the mean size of the lesions was larger; also, all the patients had breast masses as their prominent symptom, which may lead to differences between the results as in the study by Wu et al [22]. According to the BI-RADS criteria [23], we subdivided category 4 into categories 4A, 4B, and 4C, and the threshold was set to category 4A in grayscale ultrasound; consequently, the specificity was high and the sensitivity was relatively low. Moreover, S-Detect provides the final assessment in a dichotomized form of possibly benign and possibly malignant; we consider that these factors may also affect the diagnostic capability of readers combined with CAD.

### Strengths and Prospects

**Figure 3:**
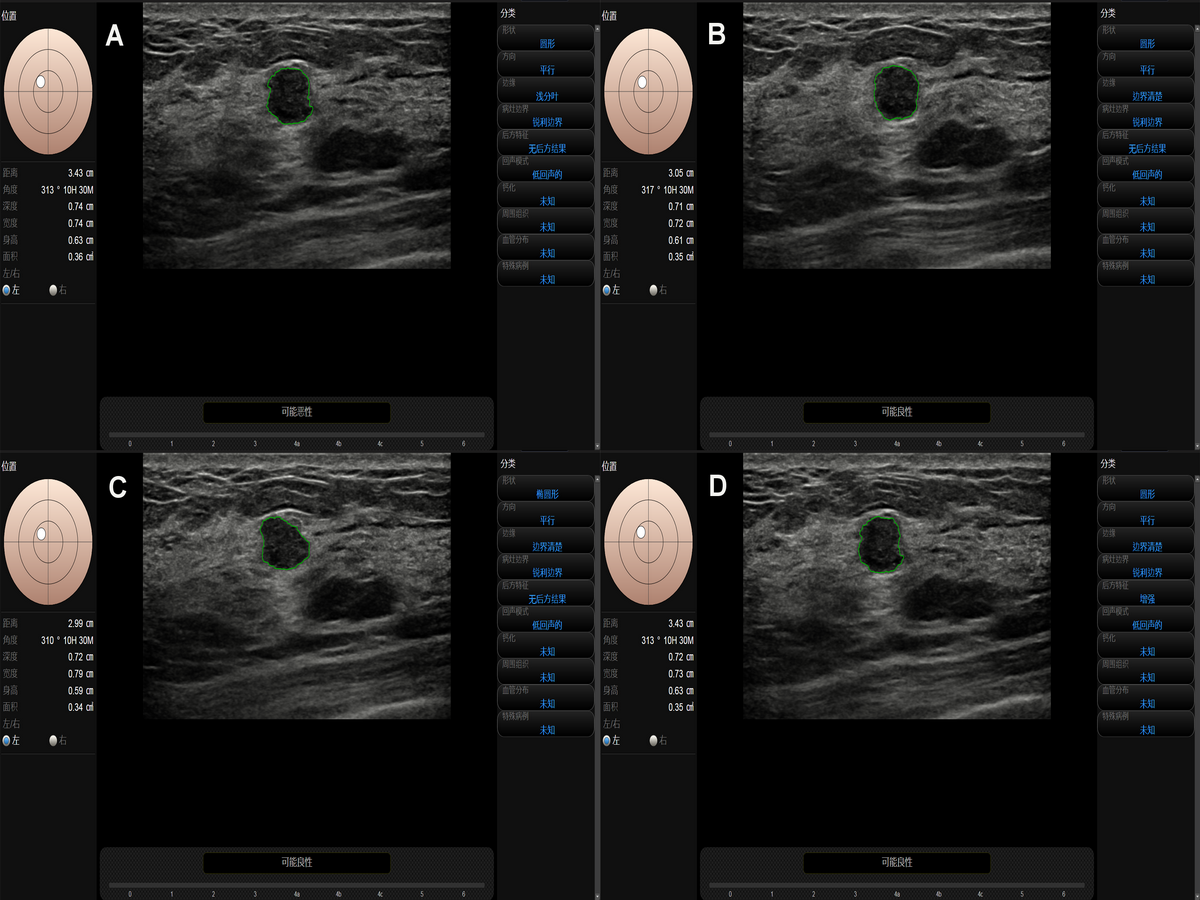
A breast lesion assessed by CAD with the cross-planes method (A, B) and the quadri-planes method (A-D).

**Figure 4:**
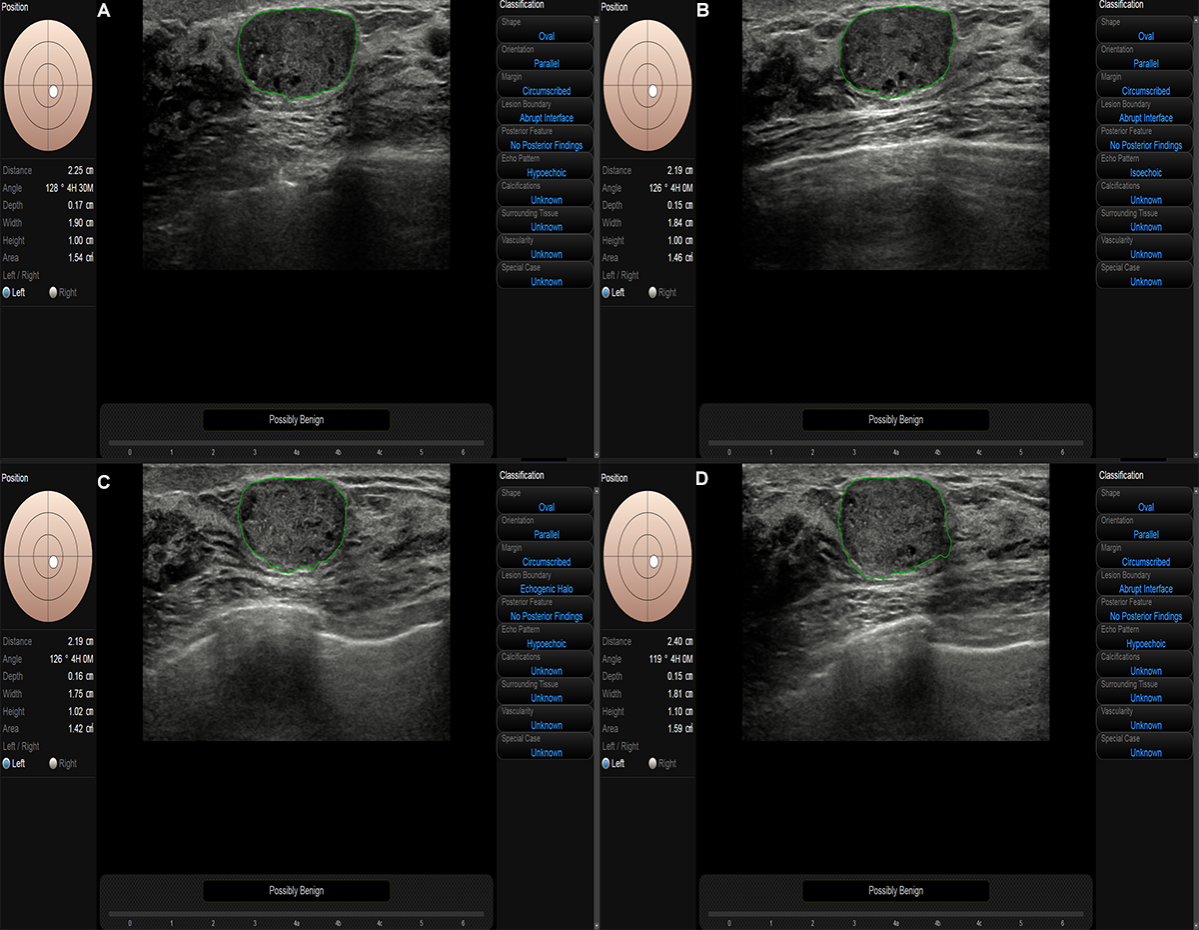
Example of a ductal carcinoma in situ lesion with a size of 1.90×1.10 centimeters showing a clear margin, regular shape, and microcalcification that was incorrectly diagnosed as benign by S-Detect with the cross-planes (A, B) and quadri-planes (A-D) methods. The lesion was classified as BI-RADS category 4B by the readers.

The results of our study are encouraging for daily clinical breast cancer screening practiced by readers, although some pathology subtypes of breast cancer had better outcomes in situ [24]. However, breast cancer is still a relatively aggressive disease that possesses higher rates of metastases and poorer survival rates [25]. Thus, it is important to detect breast cancer accurately in early stages to reduce its mortality rate [26]. Additionally, S-Detect is a concise and user-friendly program that is integrated in the ultrasound machine; the quadri-planes method enables the reader to immediately achieve a more precise result during real-time ultrasonography, which can easily be applied to routine work (Figure 3). However, it is not recommended to apply CAD alone or as a replacement for a human reader in the diagnosis of breast lesions at present, as shown in Kim’s study [6] (Figure 4). However, there is reason to believe that this will be possible in the near future. Further investigation with technical advances can be anticipated to develop a more sophisticated algorithm using the multiple plane assessment BI-RADS ultrasonographic categories.

Ultrasound scanning is a real-time and multi-angle inspection process; a lesion can be observed from different planes to collect imaging features such as the internal situation, the relationship of the lesion with its surroundings, the blood supply model, and patient histories. Obviously, ultrasound can obtain more image data and clinical information than CAD. The quadri-planes method with CAD can extract more features from a tumor with maximum objectivity; combined with the expertise of a reader, the weaknesses of each method can be counteracted by the strengths of the others, which can assist readers in making more accurate diagnoses regardless of their experience.

### Limitations

There are several limitations of this study. First, the number of cases in the single center study was relatively small. Second, the presentation of calcifications is still not included in the current version of S-Detect due to its limited analysis ability for microcalcifications [27]. Third, some small nodules classified as BI-RADS category 2 or 3 with sizes of around 1 cm without surgical operation were not included in this study, which may have affected the results. Fourth, the number of planes of the lesions for CAD was set to 4. It can be argued that it would have been better to study additional planes. Fifth, both of the readers were relatively inexperienced breast scan readers. In China, the specialty of breast imaging is new, and its staff are young compared with those in other imaging specialties. These factors may have affected the results.

### Conclusion

S-Detect is a feasible diagnostic tool that can improve the sensitivity, accuracy, and AUC of both novice and experienced readers in the quadri-planes method while also improving the specificity for the novice reader; thus, it demonstrates important application value in the clinical diagnosis of breast cancer.

## Abbreviations

AUC: area under the receiving operating characteristic curve
BI-RADS: Breast Imaging Reporting and Data System
CAD: computer-aided diagnosis
NPV: negative predictive value
PPV: positive predictive value
ROC: receiver operating characteristic
